# *Cryptosporidium cuniculus* and *Giardia duodenalis* in Rabbits: Genetic Diversity and Possible Zoonotic Transmission

**DOI:** 10.1371/journal.pone.0031262

**Published:** 2012-02-17

**Authors:** Weizhe Zhang, Yujuan Shen, Rongjun Wang, Aiqin Liu, Hong Ling, Yihong Li, Jianping Cao, Xiaoyun Zhang, Jing Shu, Longxian Zhang

**Affiliations:** 1 Department of Parasitology, Harbin Medical University, Harbin, Heilongjiang, China; 2 Key Laboratory of Parasite and Vector Biology, National Institute of Parasitic Diseases, Chinese Center for Disease Control and Prevention, Ministry of Health, World Health Organization, Collaborating Centre for Malaria, Schistosomiasis and Filariasis, Shanghai, China; 3 College of Animal Science and Veterinary Medicine, Henan Agricultural University Zhengzhou, Henan, China; Technion-Israel Institute of Technology Haifa 32000 Israel., Israel

## Abstract

**Background:**

*Cryptosporidium* and *Giardia* are the two important zoonotic pathogens causing diarrhea of humans and animals worldwide. Considering the human cryptosporidiosis outbreak and sporadic cases caused by *C. cuniculus*, the important public health significance of *G. duodenalis* and little obtained information regarding rabbit infected with *Cryptosporidium* and *Giardia* in China, the aim of this study is to determine the prevalence and molecularly characterize *Cryptosporidium* and *Giardia* in rabbits in Heilongjiang Province, China.

**Methodology/Principal Findings:**

378 fecal samples were obtained from rabbits in Heilongjiang Province. *Cryptosporidium* oocysts and *Giardia* cysts were detected using Sheather's sugar flotation technique and Lugol's iodine stain method, respectively. The infection rates of *Cryptosporidium* and *Giardia* were 2.38% (9/378) and 7.41% (28/378), respectively. Genotyping of *Cryptosporidium* spp. was done by DNA sequencing of the small subunit rRNA (SSU rRNA) gene and all the nine isolates were identified as *Cryptosporidium cuniculus*. The nine isolates were further subtyped using the 60-kDa glycoprotein (gp60) gene and two subtypes were detected, including VbA32 (n = 3) and a new subtype VbA21 (n = 6). *G. duodenalis* genotypes and subtypes were identified by sequence analysis of the triosephosphate isomerase (TPI) gene. The assemblage B (belonging to eight different subtypes B-I to B-VIII) was found in 28 *G. duodenalis*-positive samples.

**Conclusions/Significance:**

The rabbits have been infected with *Cryptosporidium* and *Giardia* in Heilongjiang Province. The results show that the rabbits pose a threat to human health in the studied areas. Genotypes and subgenotypes of *C. cuniculus* and *G. duodenalis* in this study might present the endemic genetic characterization of population structure of the two parasites.

## Introduction

*Cryptosporidium* and *Giardia* are important intestinal protozoa found in humans and animals worldwide. Both pathogens are responsible for gastroenteritis, chronic diarrhea or even severe diarrhea, depending on the age and health of the infected hosts as well as the genetic background and infective dose of the parasites.

The majority of human *Cryptosporidium* infections are attributable to *C. hominis* and *C. parvum*. However, a number of other *Cryptosporidium* species and genotypes have also been reported at a lower frequency, including nine *Cryptosporidium* species (*C. meleagridis*, *C. felis*, *C. canis*, *C. muris*, *C. suis*, *C. ubiquitum*, *C. cuniculus*, *C. fayeri* and *C. andersoni*) and five genotypes (skunk genotype, chipmunk I genotype, horse genotype, monkey genotype and pig genotype II) [1]–[8]. Although the first report of rabbit *Cryptosporidium* was noticed in 1912 [9], the concerns regarding *Cryptosporidium* infection in rabbits have only occurred in recent years due to a few sporadic human cases and a serious waterborne outbreak of cryptosporidiosis caused by *C. cuniculus* (previously named as *Cryptosporidium* rabbit genotype) [4], [10], [11]. In addition, although several studies have reported the natural infection of *C. cuniculus* in rabbits [12], few large-scale studies were available for the prevalence of *C. cuniculus* in rabbits and only a small number of studies have been conducted on genetic analysis [10], [13]–[17].

For *G. duodenalis*, seven *G. duodenalis* assemblages (A to G) are defined based on genetic analysis and host specificity. More recently, assemblage H has been identified in marine vertebrates [18]. Among which, only assemblages A and B are human pathogens and assemblage A is further classified into two major subtypes, AI and AII. Many subtypes are present in the assemblage B due to its high degree of genetic polymorphism. In contrast, assemblages C to G are mostly found in livestock, companion animals and rodents [5], [19]–[21]. Previously, most studies focused on the prevalence and molecular identification of *G. duodenalis* in livestock and wild animals. Thus far, only one *G. duodenalis* isolate from a rabbit has been identified as assemblage B based on TPI gene [20].

In China, a few studies have reported the prevalence of *Cryptosporidium* spp. in rabbits (Table 1), and the molecular identification was just seen in a more recent study [17]. In contrast, only one *G. duodenalis* isolate has been obtained from a rabbit; however, it was not genotyped and subtyped [22]. Thus, the prevalence, distribution and genetic characterization of *C. cuniculus* and *G. duodenalis* in rabbits in China are still unclear. In this study, to better understand the prevalence and transmission of cryptosporidiosis and giardiasis, an epidemiologic investigation of two parasites was conducted in rabbits in Heilongjiang province, China; further, the positive isolates of *C. cuniculus* and *G. duodenalis* were analyzed for genetic characterization, respectively.

**Table 1:** Prevalence of *Cryptosporidium* in rabbits in China.

Location	Detection method	No. positive/No. examined (%)	*Cryptosporidium* spp. and subtypes	Ref
Henan	Sucrose flotation and modified acid-fast stain	37/1081 (3.42)	*C. cuniculus*, VbA29, VbA35, VbA36	[17]
Jilin	Acid-fast stain	97/287 (33.80)	*C. parvum* *	[38]
	RAPD-PCR	1/35 (2.86)	*C. parvum* *	[39]
Shandong	Acid-fast stain	11/69 (15.94)	Not done	[40]
Heilongjiang	Sucrose flotation	9/378 (2.38)	*C. cuniculus*, VbA21, VbA32	This study

*Previous *C. parvum* identified in rabbits is currently considered to be *C. cuniculus*.

## Materials and Methods

### Ethical Considerations

Before beginning work on the study, we contacted the farm owners and obtained their permission. No specific permits were required for the described field studies. We directly collected the fecal samples instead of operating on the rabbits in this study. Each of the experimental rabbits was fed alone in each cage and the labeled plastic bags were put under each of the cages. One day later, we collected the rabbit feces excreted in the bags. During the procedure, the rabbits were not hurt at all. And the locations where we sampled are not privately-owned or protected in any way. The field studies did not involve endangered or protected species.

### Sample Collection

A total of 378 fresh fecal samples were collected from 4-6-month-old experimental rabbits on eight farms in Heilongjiang Province between October 2008 and August 2010. The Sheather's sugar flotation technique and Lugol's iodine stain method were used to detect the *Cryptosporidium* oocysts and *Giardia* cysts, respectively. Wet smears were examined using a bright-field microscope with 100× and 400× magnification. *Cryptosporidium*-positive and *G. duodenalis*-positive samples were stored in 2.5% potassium dichromate solutions at 4°C prior to DNA extraction, respectively.

### DNA extraction

Fecal samples were washed twice with distilled water, and genomic DNA was extracted using a QIAamp DNA Stool Mini Kit (QIAgen, Hilden, Germany) according to the manufacturer's instructions. DNA was eluted in 200 µL of Buffer AE and stored at −20°C prior to use in PCR analysis.

### *Cryptosporidium* genotyping and subtyping

*Cryptosporidium* oocysts in the samples were identified to the species/genotype level using nested PCR amplification of an approximately 830 bp fragment of the SSU rRNA gene [23]. Subtyping of *Cryptosporidium*-positive samples was done by nested PCR amplification of an approximately 800–850 bp fragment of the gp60 gene [24]. All secondary PCR products of both genes were sequenced using the secondary PCR primers.

### *G. duodenalis* genotyping

The identity of *G. duodenalis* genotypes and subtypes were determined by DNA sequence analysis of the TPI gene. *G. duodenalis*-positive samples were used to amplify the partial TPI gene of an approximately 530 bp fragment by a nested PCR [20]. Genotype and subtype identities of the *G. duodenalis* samples were established by direct comparison of the acquired sequences with reference sequences downloaded from GenBank.

### Sequence analysis

All secondary PCR products were sequenced in both directions using the secondary PCR primers on an ABI PRISMTM 3730 XL DNA Analyzer (Applied Biosystems, USA) by using the BigDye Terminator v3.1 Cycle Sequencing Kit (Applied Biosystems). Nucleotide sequences obtained were aligned with *Cryptosporidium* and *G. duodenalis* reference sequences from GenBank using ClustalX 1.81. The representative nucleotide sequences obtained in the study were deposited in the GenBank database under the following accession numbers: HQ397716 to HQ397718 (*Cryptosporidium*) and HQ397719, HQ666892 to HQ666898 (*G. duodenalis*).

## Results

### Prevalence of *Cryptosporidium* and *Giardia* in rabbits

A total of 378 fecal samples were examined by microscopy. Nine samples were positive for *C. cuniculus* (2.38%), and 28 samples were positive for *G. duodenalis* (7.41%). And *G. duodenalis* was more widespread than *C. cuniculus*, accounting for *C. cuniculus* oocysts found in four farms and *G. duodenalis* cysts found in seven farms. Both *Cryptosporidium* and *Giardia* were detected on Farms 2, 3, 4 and 8 (Table 2). Additionally, one mixed infection with both parasites was found on Farm 3.

**Table 2:** Prevalence and subtype distribution of *C. cuniculus* and *G. duodenalis* in rabbits in Heilongjiang Province, China.

Farm	Sample size	*C. cuniculus*		*G. duodenalis*	
		No. of positive (%)	Subtype (No.)	No. of positive (%)	Subtype (No.)
Farm1	66	0 (0)		0 (0)	0
Farm2	72	0 (0)		5 (6.94)	B-I(2), B-II(2), B-III(1)
Farm3	45	2 (4.44)	VbA21(1),VbA32(1)	7 (15.56)	B-I(6), B-IV(1)
Farm4	76	3 (3.95)	VbA21(2),VbA32(1)	7 (9.21)	B-I(5), B-V(1), B-VI(1)
Farm5	36	2 (5.56)	VbA21(2))	2 (5.56)	B-I(1), B-VII(1)
Farm6	12	0 (0)	VbA21(1),VbA32(1)	3 (25.00)	B-I(2), B-VII(1)
Farm7	18	0 (0)		1 (5.56)	B-I(1)
Farm8	53	2 (3.77)		3 (5.66)	B-I(1), B-VIII(2)
Total	378	9 (2.38)		28 (7.41)	

### *Cryptosporidium* genotyping and subtyping

DNA sequencing of the SSU rRNA PCR products showed that all the nine sequences were identical to *C. cuniculus* isolates from China, the UK, the Czech Republic and Australia [4], [10], [13], [14], [16], [17]. Subtyping was further achieved by sequence analysis of the gp60 gene. Two subtypes were identified in the nine *C. cuniculus*-positive isolates: VbA21 (n = 6) and VbA32 (n = 3).

### *Giardia* genotyping and subtyping

Alignment of the TPI sequences indicated that 28 *G. duodenalis*-positive isolates all belonged to assemblage B and represented eight distinct subtypes, B-I to B-VIII (Table 2). Subtype B-I was identical to a rabbit-derived isolate (AY228639) and it was the most common subtype found in this study, accounting for 64.3% (18/28) of *G. duodenalis*-positive samples. In contrast, subtypes (B-II to B-VIII) were not identical to any known assemblage B subtypes with each subtype having only one or two cases. By using the GenBank sequence AY368171 as a reference sequence, single nucleotide polymorphisms were present in the eight representative sequences obtained from the 28 *G. duodenalis*-positive isolates and one to three base variations were found (Table 3).

**Table 3:** Variation in the TPI nucleotide sequences among subtypes of *G. duodenalis* assemblage B in rabbits in Heilongjiang Province.

Subtype	No. of isolates	Percentage (%)	Nucleotide at position							Accession no. in GenBank
			30	43	144	199	229	374	388	
Ref sequence			A	C	G	G	T	A	A	AY368171
B-I	18	64.29	A	C	G	A	T	A	A	HQ397719
B-II	2	7.14	A	C	G	A	T	A	G	HQ666892
B-III	1	3.57	C	C	G	A	T	A	A	HQ666893
B-IV	1	3.57	A	A	G	A	T	A	A	HQ666894
B-V	1	3.57	A	A	A	A	T	A	A	HQ666895
B-VI	1	3.57	A	C	G	A	T	G	A	HQ666896
B-VII	2	7.14	C	A	G	A	T	A	A	HQ666897
B-VIII	2	7.14	A	C	G	G	C	A	A	HQ666898

## Discussion

A 2.38% overall infection rate of *Cryptosporidium* spp. was observed in this study, which was lower than those (2.86% to 33.80%) in rabbits conducted in the other areas in China (Table 1). It was also not as high as those in Japan (19.7%; 13/66) and Australia (6.8%; 12/176) [15], [16]. The differences in prevalence may be related to the factors such as the sensitivity and specificity of detection methods, animal health at the time of sampling, the experimental design and the overall sample size. And health status of animals is closely associated with the animal age. *Cryptosporidium* is often found in unhealthy juvenile rabbits. A report showed that dead juvenile rabbits had a higher infection rate than healthy rabbits (19.7% vs 3.33%) [15]. In addition, *Cryptosporidium* infections in neonatal and younger rabbits are associated with high mortality and diarrheic feces. Just like *C. parvum* in cattle, there appears to be a decline in *Cryptosporidium* prevalence, symptoms and oocysts shedding as rabbits increase in age [12], [17], [25]. The lower prevalence in this study might result from our fecal sampling of healthy older rabbits ranging from four to six months of age.

Sequence analysis of SSU rRNA gene showed that the nine isolates shared 100% similarity to most of *C. cuniculus* cases from humans and rabbits although there were two nucleotide differences compared to a New Zealand isolate (AY458612) [4], [10], [11], [13], [14], [16], [17], [26]. Thus, the narrow host spectrum of *C. cuniculus* further supports previous theory that there is an apparent host adaptation and parasite-host co-evolution in *Cryptosporidium* [13]. Gp60 gene subtyping revealed the existence of two subtypes in this study, VbA21 and VbA32. To date, at least 20 subtypes from humans and 9 subtypes from rabbits have been identified (Table 4). VbA32 has been isolated from humans in the UK [27]. In contrast, VbA21 was not identical to any known subtypes, thus representing a new subtype. It is unclear about the host specificity of the two subtype families Va and Vb. Va subtype family is mostly found in humans with occasionally seen in rabbits [10], [27], [28]. Vb subtype family is mostly found in rabbits with commonly seen in humans in the UK [10], [16], [17], [27]. This showed that human-derived and rabbit-derived isolates of *C. cuniculus* do not have strict host specificity and cross transmission may occur between humans and rabbits. The subtypes in this study might have the potential to infect humans, especially for the subtype VbA32 found in humans.

**Table 4:** Subtypes of *C. cuniculus* in humans and rabbits in different countries.

Country	Subtype		Ref
	Human	Rabbit	
Australia		VbA23R3,VbA26R4	[16]
China		VbA29	[10]
		VbA29,VbA35,VbA36	
		VbA21,VbA32	This study
Czech Republic		VbA19	[10]
UK	VaA18,VaA22	VaA18	[10]
	VaA9, VaA11, VaA18, VaA19, VaA21,VaA22, VbA20, VbA22, VbA25, VbA26,VbA28, VbA29,VbA30, VbA32, VbA33, VbA34, VbA36,VbA37		[27]
	VaA18,VaA22, VaA23, VaA32,		[28]

For *G. duodenalis*, only one case of rabbit giardiasis caused by assemblage B has been reported [20]. In this study, a 7.41% infection rate of *Giardia* was noticed, which was higher than the 2.38% prevalence of *Cryptosporidium*. Sequence analysis of TPI gene showed that all the isolates belonged to *G. duodenalis* assemblage B and represented eight different subtypes (B-I to B-VIII) with B-I as the dominant subtype(64.3%, 18/28). Among which, only subtype B-I was identical to a previous rabbit-derived isolate (AY228639), whereas the remaining subtypes have never reported. Previously, the diversity of *G. duodenalis* assemblage B has also been observed in isolates from humans and other animals [5]. In a recent study in China, the polymorphism of assemblage B is also expected that six human isolates belonged to six distinct subtypes [29]. Unlike assemblage A, the nomenclature of assemblage B subtype was relatively disordered, which might cause trouble in understanding the transmission of *G. duodenalis* assemblage B. Thus, it is necessary for the researchers to make a uniform rule to name different subtypes of assemblage B in the future.

*G. duodenalis* assemblage B has a broad range of host. Except for having been found in humans, it has also been detected in cattle sheep, horses, dogs, and cats [30]–[35]. So far, although no strong evidence has supported the zoonotic transmission of *G. duodenalis* between humans and animals, case control studies showed that contacting with farm animals was related to the increasing infection rates of giardiasis [36], [37].

In conclusion, the present study provided useful data for further studying the *C. cuniculus* and *G. duodenalis* infections in rabbits at prevalence rates and molecular levels. Based on the fact that both parasites all belonged to zoonotic pathogens, more extensive studies in rabbits in different areas are needed to better characterize the transmission of cryptosporidiosis and giardiasis and to assess the public health significance of these parasites.
